# Case Report: Abdominal pseudohernia preceding the herpes rash: a pre-eruptive diagnostic pitfall in herpes zoster

**DOI:** 10.3389/fmed.2026.1975268

**Published:** 2026-10-15

**Authors:** Binbin Huang, Jia Lin, Xiaomo Wu

**Affiliations:** 1 Department of Integrated Traditional Chinese and Western Medicine Dermatology, Fuzhou Dermatology Hospital, Fuzhou, China; 2 Regenerative Medicine and Gene Therapy Lab, Fuzhou Dermatology Hospital, Fuzhou, China; 3 Department of Biomedicine, University of Basel, Basel, Switzerland

**Keywords:** herpes zoster, abdominal pseudohernia, segmental zoster paresis, lamp-fire moxibustion, varicella-zoster virus, diagnostic pitfall

## Abstract

Herpes zoster is usually recognized by its painful dermatomal eruption, although its motor complications are often overlooked. Herpes zoster–associated abdominal pseudohernia is a rare segmental motor manifestation in which paresis of the abdominal wall muscles produces a unilateral bulge without a true fascial defect. In most patients with herpes zoster, the bulge develops 1 to several weeks after the rash; in a minority of patients, it precedes the eruption, creating a particular risk of misdiagnosis. Herein, we describe a 66-year-old man with controlled diabetes and hypertension who developed a painful left abdominal bulge 4 days before the appearance of skin lesions. Routine laboratory tests were unremarkable, and abdominal computed tomography results showed no fascial defect or alternative structural cause of the bulge. Four days after the appearance of the bulge, erythema and grouped vesicles developed over the left T10–T12 dermatomes without crossing the midline. Before the dermatology visit, the patient had undergone lamp-fire moxibustion of the affected area as a folk remedy, after which the lesions rapidly crusted and the patient developed extensive postinflammatory hyperpigmentation. No fresh vesicles were present at dermatologic examination. A presumptive clinical diagnosis of herpes zoster–associated abdominal pseudohernia was made based on the segmental abdominal bulge, dermatomal distribution of the skin changes, and the reported history of a vesicular eruption. With conservative management, the bulge markedly regressed within 3 months. This case highlights that a new unilateral segmental abdominal bulge, even when it precedes the characteristic rash, should prompt consideration of herpes zoster to avoid unnecessary surgical intervention.

## Introduction

Herpes zoster (HZ) results from the reactivation of latent varicella-zoster virus (VZV) in the dorsal root ganglia and is characterized by unilateral dermatomal pain and a vesicular eruption (1). Motor involvement is less commonly recognized but can be clinically significant. Segmental zoster paresis involving the abdominal wall causes localized weakness of the abdominal musculature, producing an outward bulge that resembles a true hernia but occurs in the absence of a fascial defect; this condition is termed herpes zoster–associated abdominal pseudohernia. Abdominal muscle paralysis associated with HZ was first reported in the late 19th century and remains an uncommon complication (2). Motor paresis has been reported in approximately 0.5%–5% of patients with HZ, with abdominal wall involvement representing an even rarer subset (3–5). The virus may spread from the sensory ganglion to the anterior horn cells, adjacent ventral motor roots, or peripheral motor nerves, resulting in segmental weakness of the ipsilateral abdominal musculature (3, 6). In most patients with HZ, the abdominal bulge develops 1–8 weeks after the cutaneous eruption, and by this time, the preceding rash usually provides an important diagnostic clue (3). However, in approximately 10% of patients with HZ and motor paresis, the bulge precedes the eruption (5). In the absence of characteristic skin lesions, the presentation may be mistaken for an incisional, Spigelian, or lumbar hernia, potentially leading to repeated or unnecessary imaging and, in some patients, surgical exploration (7). Herein, we report a patient in whom abdominal pseudohernia preceded the cutaneous eruption and highlight the diagnostic challenges of this pre-eruptive presentation.

## Case description

This case report was prepared and is reported in accordance with the CARE guidelines for case reports. A 66-year-old man presented to the dermatology department with a 10-day history of a painful bulge on the left side of the abdomen. He had type 2 diabetes mellitus and hypertension, both of which were managed with oral medications, and had no history of abdominal surgery or trauma. Ten days before the dermatology visit, the patient had been evaluated at an outside hospital for the same abdominal bulge. A complete blood count and routine biochemical test results were unremarkable. A whole-abdomen computed tomography (CT) scan showed an intact abdominal fascia, no intra-abdominal mass, and no appreciable thinning or asymmetry of the ipsilateral abdominal wall musculature. Thus, CT excluded a true hernia but did not identify an alternative structural cause of the bulge. Furthermore, no virological confirmation was obtained; VZV PCR of a crust or a lesion swab, a Tzanck smear, and paired serology were not performed, and electromyography was also not performed.

Approximately 4 days after the first appearance of the abdominal bulge (6 days before the dermatology visit), erythema developed over the left abdomen, followed by grouped, pruritic, and painful vesicles. On dermatologic examination, large areas of postinflammatory hyperpigmentation in a band-like distribution were present over the left lumbodorsal region, with residual lesions and scattered dark-red papules seen within the affected area. The lesions were confined to the left side and did not cross the posterior midline. A localized soft bulge was observed over the ipsilateral anterior abdominal wall. The overlying skin showed faint erythema and scattered residual papules without fresh vesicles, erosions, or skin breakdown (Figure 1). Notably, between the onset of the vesicular eruption and the dermatology visit, the patient had undergone lamp-fire moxibustion (denghuo moxibustion) of the affected skin as a folk remedy. This thermal intervention may have contributed to rapid crusting and extensive postinflammatory hyperpigmentation, explaining the absence of fresh vesicles at presentation. Because no fresh vesicles were present at the time of examination and no virological or electrophysiological confirmation was obtained, a presumptive clinical diagnosis of herpes zoster–associated abdominal pseudohernia was made based on the segmental abdominal bulge, absence of a fascial defect, dermatomal distribution of the skin changes, and reported history of a vesicular eruption.

**Figure 1:**
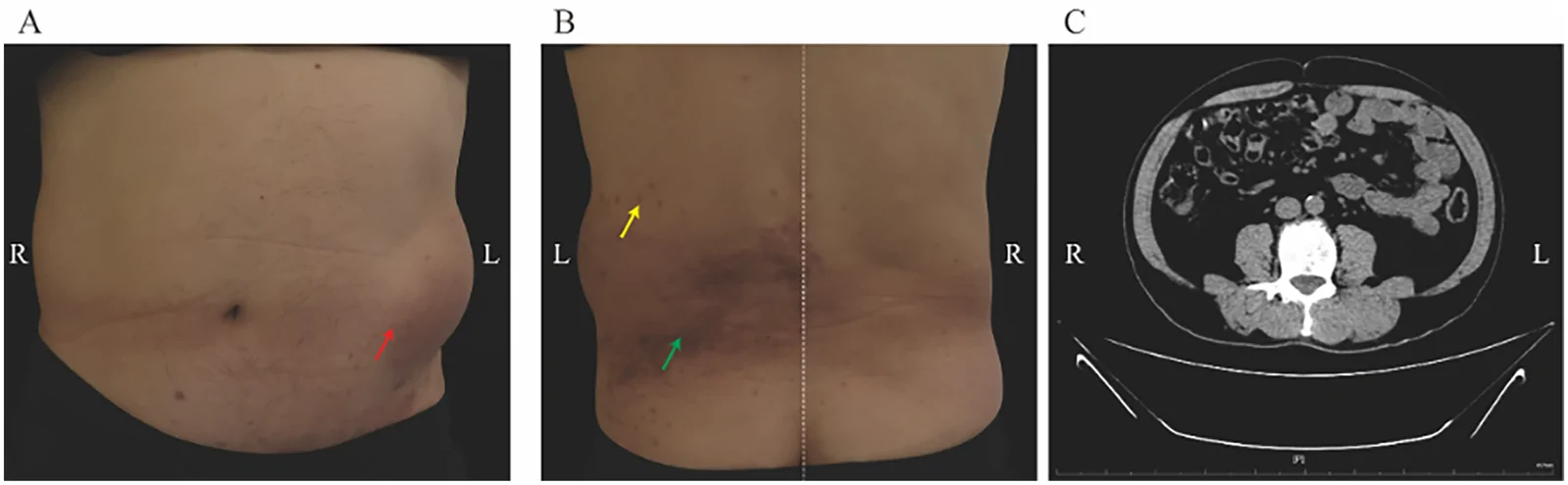
Clinical and computed tomography findings. **(A)** The left abdominal wall bulge before treatment (indicated by the red arrow). **(B)** Unilateral band-shaped postinflammatory hyperpigmentation in the lumbodorsal region with residual lesions and scattered dark-red papules. The yellow arrow indicates hyperpigmentation at the site of lamp-fire moxibustion, and the green arrow indicates postinflammatory hyperpigmentation associated with herpes zoster eruption. **(C)** Abdominal CT showing an intact abdominal wall fascia with no hernia or intra-abdominal mass.

The patient was administered oral mecobalamin (0.5 mg three times daily), gabapentin (0.1 g twice daily), and neurotropin injection (3 mL once every 2 days) and was advised to avoid strenuous exertion. No surgical intervention was undertaken. At follow-up after approximately 3 months, the abdominal bulge had markedly regressed compared with baseline. The clinical course is summarized in Table 1.

**Table 1:** Timeline of the clinical course.

Time point	Clinical event
Day 0 (approximately 10 days before the dermatology visit)	A new painful bulge was observed in the left abdomen. Observations at an outside hospital: routine blood tests and biochemistry were unremarkable, and the whole-abdomen CT scan showed an intact abdominal fascia, no intra-abdominal mass, and no appreciable thinning or asymmetry of the ipsilateral abdominal wall musculature. No alternative structural cause was identified.
Day 4 (approximately 6 days before the dermatology visit)	Erythema followed by grouped, pruritic, and painful vesicles appeared on the left abdomen. The patient received lamp-fire moxibustion (denghuo moxibustion) as a folk therapy.
Day 10 (dermatology visit)	On the left trunk within the T10–T12 dermatomes, large band-shaped postinflammatory hyperpigmented patches were visible over the lumbodorsal region, with residual traces of old lesions and scattered dark-red papules. The lesions were confined to one side of the body and did not cross the posterior midline. A localized soft bulge was observed in the ipsilateral anterior abdominal wall. The overlying abdominal skin revealed faint erythema and scattered old papules without fresh vesicles, erosions, or skin breakdown. The patient complained of severe pain. Presumptive clinical diagnosis: herpes zoster–associated abdominal pseudohernia
Day 10 onward	Mecobalamin oral (0.5 mg three times daily), gabapentin capsules (0.1 g twice daily), and neurotropin injection (3 mL once every 2 days) were administered. The patient was advised to avoid strenuous exertion.
Approximately 3 months	The abdominal bulge was markedly reduced compared with the baseline.

CT, computed tomography.

## Discussion

This case highlights the diagnostic difficulty of herpes zoster–associated abdominal pseudohernia when abdominal wall weakness precedes the characteristic skin eruption. Herpes zoster–associated abdominal pseudohernia is a rare segmental motor manifestation of HZ. Weakness of the oblique and transversus abdominis muscles reduces the muscular support of the abdominal wall, allowing the abdominal contents to displace the weakened wall outward and produce a localized bulge that resembles a true hernia despite an intact fascia (3, 6). The temporal relationship between the abdominal bulge and the cutaneous eruption can complicate recognition of this condition. In most patients with HZ and skin eruption, the bulge develops 1–8 weeks after the eruption (3). Our patient exhibited the less common sequence in which the bulge appeared first, which was approximately 4 days before the vesicular eruption. This pre-eruptive presentation, reported in approximately 10% of cases with motor paresis, poses a particular diagnostic challenge because the characteristic dermatomal eruption that would suggest HZ has not yet appeared (5).

Several clinical features in our patient supported the presumptive diagnosis of HZ-associated abdominal pseudohernia. First, the abdominal bulge was unilateral and segmental, which corresponded to the T10–T12 distribution of the subsequent cutaneous eruption. Second, CT imaging demonstrated an intact abdominal fascia and excluded a true hernia and intra-abdominal mass. In HZ-associated abdominal pseudohernia, CT or ultrasonography results typically demonstrate an intact fascia and may additionally reveal thinning or asymmetry of the affected abdominal wall musculature rather than a structural defect (5). On targeted re-evaluation of the CT images in our patient, no appreciable thinning or asymmetry of the ipsilateral abdominal wall musculature was observed, and therefore, imaging was primarily useful for excluding structural causes rather than providing positive radiological evidence for the diagnosis. Magnetic resonance imaging may be useful when spinal or nerve root compression needs to be excluded. Third, the abdominal bulge markedly regressed within 3 months with conservative management, a clinical course consistent with the generally favorable recovery described in HZ-associated segmental motor paresis.

Given the patient's history of type 2 diabetes mellitus, diabetic thoracic radiculopathy (truncal radiculoneuropathy) was an important differential diagnosis. This condition can also present with unilateral truncal pain and segmental abdominal wall weakness or bulging, particularly in patients with diabetes (8). In our patient, the subsequent development of a unilateral dermatomal vesicular eruption supported HZ as the more likely clinical explanation. Other structural causes of abdominal wall bulging, including incisional, Spigelian, and lumbar hernias and intra-abdominal masses, were not supported by the imaging findings.

Risk factors for HZ and its complications, including advanced age, diabetes mellitus, immunosuppression, and underlying malignancy, are relevant to clinical assessment and counseling (1). Our patient was an older adult with diabetes mellitus. Antiviral therapy was not initiated because he presented to the dermatology department 10 days after the symptom onset, during which no fresh vesicles were present and the usual early treatment window had passed (1). The atypical appearance of the skin lesions, which were largely replaced by hyperpigmented patches after moxibustion, also complicated recognition of the preceding vesicular eruption.

This case also illustrates an underreported confounding factor: the application of folk thermal remedies to active zoster lesions. Lamp-fire moxibustion involves direct or near-contact heat to the skin and may alter the morphology of vesicular lesions, contributing to crusting and postinflammatory hyperpigmentation that can obscure the characteristic grouped vesicles (9). Clinicians should be aware that atypical dermatomal lesions may reflect a prior moxibustion or other heat-based folk therapies rather than an intrinsically atypical zoster eruption. They should specifically inquire about such practices to avoid delays in diagnosis. Gabapentin and neurotropin were administered for neuropathic pain control. Neurotrophic agents, including mecobalamin, are commonly added to the treatment regimen, although evidence supporting their capacity to fasten motor recovery is limited (10). Surgical intervention is generally unnecessary once a true structural hernia has been excluded because most pseudohernias resolve spontaneously over several months, although residual weakness may persist in a minority of patients. Table 2 summarizes the present case alongside representative reports, showing the predominance of conservative management and generally favorable outcomes.

**Table 2:** Selected reported cases of herpes zoster–associated abdominal pseudohernia.

Reference	Patient(s) and segment	Bulge vs. rash sequence	Management	Outcome
Present case (2026)	66/M, left T10–T12	Bulge preceded rash by approximately 4 days	Gabapentin, neurotropin, and mecobalamin; antiviral not administered (late presentation with no fresh vesicles)	Marked reduction at 3 months
Postherpetic pseudohernia review, *Cureus* 2025 (4)	68/M, unilateral thoracic abdominal wall; with a review of 94 reported cases of patients	Paresis approximately 2 weeks after completing antiviral therapy	Pregabalin plus vitamin B12	Improved within 2 months
Chiew and Pawa, *Heliyon* 2023 (5)	34 pooled cases of patients; median age, 71.5 years; T11 most frequently affected	Pre-eruptive in 4 of 34 (12.5%)	Supportive	Good recovery in most; a median follow-up of 3 months
Yoo et al., *JAAD Case Rep* 2019 (11)	3 patients; thoracoabdominal	Posteruptive; in one case, the bulge appeared approximately 1 day after the vesicles	Supportive	Resolution
Shastry et al., *Indian Dermatol Online J* 2021 (12)	3 patients; lower thoracic	Posteruptive; 10–15 days after the vesicles in all three patients	Supportive	Resolution
Tirelli et al., *Int J Dermatol* 2019 (13)	1 patient plus review	Posteruptive	Supportive	Self-limited
Hashimoto et al., *Intern Med* 2021 (14)	80/M with dementia; abdominal wall	Approximately 2 weeks after a rash reported only by his wife	Observation only	Deformity and CT muscle thinning both improved at 2 months

M, male.

Entries are illustrative rather than exhaustive. Approximately 94 cases have been reported in total.

This case report has several important limitations, and the diagnosis of HZ-associated abdominal pseudohernia remains presumptive. No virological confirmation was obtained: VZV PCR of the lesional material, a Tzanck smear, and paired serology were not performed. Moreover, electromyography, which can provide neurophysiological evidence of segmental denervation in affected myotomes and paraspinal muscles, was also not performed (2, 5). Consequently, the motor involvement was inferred clinically rather than being confirmed electrophysiologically. Without electromyographic evidence of denervation in the corresponding myotomes, segmental motor involvement could not be confirmed, and other potential causes of abdominal wall weakness remain possible. In addition, the vesicular stage was not observed by a physician, and no patient-taken photograph of the vesicular eruption was available. Therefore, the cutaneous eruption was documented only in patient history. Targeted re-evaluation of the CT images showed no thinning or asymmetry of the ipsilateral abdominal wall musculature, and therefore, the supportive radiological finding described in some patients with HZ was absent. Thus, the diagnosis was based on the characteristic temporal and anatomical relationship between the segmental abdominal bulge and subsequent dermatomal eruption, the absence of a structural defect on imaging, and the self-limited clinical course. These limitations should be considered when interpreting a case.

## Conclusion

To conclude, a new unilateral, segmental abdominal bulge without a fascial defect should prompt consideration of herpes zoster, even when a rash is absent or has not yet appeared. Recognizing the pre-eruptive form of abdominal pseudohernia may help avoid unnecessary surgical exploration; the condition is generally self-limited and can be managed conservatively.

## Data Availability

The original contributions presented in the study are included in the article. Further inquiries can be directed to the corresponding author.
